# Ultrasensitive and highly specific detection of the *Brucella* genus and *B*. *melitensis* by CRISPR/Cas12b‐multiple cross displacement amplification technique

**DOI:** 10.1128/jcm.01532-24

**Published:** 2025-04-11

**Authors:** Sha Mao, Xinggui Yang, Yue Wang, Fengming Chen, Hai Jiang, Yi Wang, Yingqian Kang, Shijun Li

**Affiliations:** 1 School of Public Health, the key Laboratory of Environmental Pollution Monitoring and Disease Control, Ministry of Education, Guizhou Medical University 74628, Guiyang, Guizhou, China; 2 Guizhou Center for Disease Control and Prevention 577390, Guiyang, Guizhou, China; 3 Chinese Center for Disease Control and Prevention 12415, Beijing, Beijing, China; 4 Experimental Research Center, Capital Children's Medical Center, Capital Medical University, Capital Institute of Pediatrics 36776, Beijing, Beijing, China; 5 Molecular Diagnostics Center, Capital Children's Medical Center,Capital Medical University, Beijing, China; 6 School of Basic Medical Science and Institution of One Health Research, Guizhou Medical University 74628, Guiyang, Guizhou, China; Endeavor Health, Evanston, Illinois, USA

**Keywords:** *Brucella *genus, *B*. *melitensis*, CRISPR, Cas12b, multiple cross displacement amplification

## Abstract

Brucellosis is caused by members of the *Brucella* spp. and remains one of the world’s major zoonotic diseases. *Brucella melitensis* (*B. melitensis*) as the most contagious *Brucella* species cannot be ignored as an essential source of infection for brucellosis, especially in countries/regions dominated by animal husbandry. Thus, the identification of the *Brucella* genus and *B. melitensis* is crucial for rapid diagnosis of brucellosis to control disease transmission and clinical treatment. Here, we developed the CRISPR/Cas12b nuclease combined with a multiple cross displacement amplification (MCDA) assay (CRISPR-MCDA) for highly specific and sensitive detection of *Brucella* genus and *B. melitensis* in clinical applications. Two sets of specific primers were designed targeting the specific gene of *Brucella* genus (*Bcsp31*) and *B. melitensis* (*BMEII0466*), respectively. The CRISPR-MCDA assay showed high specificity and sensitivity in 28 non-*Brucella* isolates and 64 clinical samples. The detection limit of CRISPR-MCDA assay was 2 copies/μL in the plasmid dilution template, and the whole detection process took within 90 minutes with nanoparticle-based lateral flow biosensor (LFB) to validate experimental results. Taken together, the CRISPR-MCDA-LFB assay is a visual, sensitive, and highly specific detection technique that can be used as an attractive potential identification tool for *Brucella* genus and *B. melitensis*.

**IMPORTANCE:**

The prevention and control of Brucellosis urgently require rapid and accurate diagnostic methods. This work validates a new method for the simultaneous detection of *Brucella* genus and *B. melitensis*. The method can effectively reduce the chances of contamination and provides a more rapid, sensitive, and specific on-site detection of *Brucella*. It also offers a solution for the rapid screening of Brucellosis in resource-limited environments, which is crucial for effective disease prevention and control. This technology can also be widely applied to the rapid detection of other pathogens beyond *Brucella*.

## INTRODUCTION

Brucellosis is a zoonotic infectious disease caused by *Brucella* spp. According to the World Health Organization (WHO), over 500, 000 new cases of brucellosis are reported worldwide annually. It mainly causes symptoms such as undulant fever, chronic infections, abortion, and orchitis in domestic and wild animals, directly affecting the development of animal husbandry, causing substantial economic losses, and seriously threatening human health (1–4). Importantly, there is currently no specific vaccine for human infection with *Brucella* (5). At present, the *Brucella* genus consists of six “classical” or “core” species: *B. melitensis*, *Brucella abortus* (*B. abortus*), *Brucella suis* (*B. suis*), *Brucella canis* (*B. canis*), *Brucella ovis* (*B. ovis*), and *Brucella neotomae* (*B. neotomae*) (6). Among them, *B. melitensis* is known as the most contagious *Brucella* species (7). Moreover, in the majority of laboratory acquired brucellosis cases, the serotype was reported as *B. melitensis* (8). Thus, the early, sensitive, specific, and reliable identification of the *Brucella* genus and *B. melitensis* is a key strategy to control the spread of brucellosis and facilitate clinical management and the formulation of public health measures, especially in countries/regions dominated by animal husbandry.

Currently, the canonical diagnostic tool for *Brucella* pathogens identification is the bacterial culture method. However, the lengthy detection time (typically 2–3 days), complex detection workflow, and potential risk of infection to laboratory personnel present significant challenges in this approach (3, 9, 10). In contrast, serological testing offers a rapid, cost-effective, and safer alternative with higher sensitivity, making it the preferred method in clinical practice. However, its specificity remains a significant limitation (11, 12). Molecular techniques, such as polymerase chain reaction (PCR), are also employed to detect bacteria in blood and serum samples. PCR is a highly sensitive technique that can detect low bacterial loads, making it a valuable tool for diagnosing infectious diseases. Nonetheless, the detection sensitivity of PCR exhibits significant variability, with reported ranges spanning from 50% to 100% (6, 13, 14). Furthermore, the need for specialized equipment and controlled conditions limits the feasibility of PCR-based assays for point-of-care testing (POCT). Multiple cross displacement amplification (MCDA) is an emerging isothermal amplification nucleic acid analysis tool with high sensitivity and specificity. It only requires a simple constant temperature instrument and has been successfully applied to detect many pathogens, including bacteria, viruses, and fungi (15–18). Currently, the carryover contamination in MCDA reaction is a concern; however, AUDG can effectively solve the problem of residual contamination (16, 19). Meanwhile, CRISPR can enhance the detection efficiency of MCDA and reduce the possibility of false positives caused by carryover contamination (*cis*-cleavage of the MCDA amplification products).

The CRISPR/Cas system has emerged as an efficient and versatile tool, extensively utilized in gene editing and gene regulation mechanism research (20, 21). Its rapid, sensitive, and specific characteristics make it a powerful tool for pathogen detection, single-nucleotide polymorphism (SNP) analysis, and gene mutation identification (22–24). Cas12b, a CRISPR-associated nuclease, can better identify SNPs in DNA molecules by targeting both single-stranded (ssDNA) and double-stranded (dsDNA) DNA (24). When the *trans*-cleavage activity of Cas12b nuclease is activated, the complex exhibits non-specifical and indiscriminate cleavage of nearby non-target ssDNA reporter (25, 26). Therefore, we combined CRISPR-Cas12b with MCDA reaction and validated the experimental results using a nanoparticle-based lateral flow biosensor (LFB), providing a faster, more convenient, and clinically applicable solution for field monitoring.

In this research, the CRISPR/Cas12b nuclease combined MCDA assay was an intuitive, reliable, ultrasensitive, and highly specific detection method for *Brucella* genus and *B. melitensis* (CRISPR-MCDA-LFB). The entire testing process, including DNA extraction, isothermal amplification, *trans*-cleavage of ssDNA reporter, and product validation, can be completed within 90 minutes. In addition, the detection limit of the CRISPR-MCDA-LFB assay was 2 copies/μL, and there was no cross-reactivity with other non-*Brucella* strains. The method’s practicality was evaluated using various clinical samples and compared with the *Brucella*-PCR method. The evaluation assays demonstrated that the CRISPR-MCDA-LFB method was an alternative, convenient, and specific detection method for clinical detection and POCT of brucellosis diagnosis.

## MATERIALS AND METHODS

### Materials and instruments

The amplification reagent (10 × reaction buffer, dATP, dUTP, dGTP, dCTP, MgSO_4_, *Bst* 8.0 DNA polymerase) for MCDA and AUDG were provided by Beyotime Biotechnology Co., Ltd. (Shanghai, China). The DNA extraction kits (QIAamp DNA min, QIAGEN) were purchased from Kai Jie Technology Development Co., Ltd. (Shanghai, China). AapCas12b nuclease (c2c1, 10 pmol/mL) purchased from Shanghai HuicH Biotech Co., Ltd. (Shanghai, China). Gold nanoparticle-based LFB purchased from Wobo Biotechnology Co., Ltd. (Nanjing, China). MG chromogenic reagents were obtained from HuiDeXin Biotechnology (Tianjin, China). PCR kit provided by Takara Co., Ltd. (Beijing, China). A real-time turbidity reader (LA-500) was provided by Eiken Chemical Co., Ltd. (Japan). The ChemiDoc MP imaging system was obtained from Bio-Rad (USA), and the Qubit4 Fluorometer system was obtained from Thermo Fisher Scientific (Waltham, MA). Gentier 96E/96R fully automatic medical PCR analysis system purchased from Tianlong Technology Co., Ltd (xian, China).

### Bacterial culture and PCR method

The sample was inoculated into a biphasic blood culture bottle and *Brucella* was cultured in a CO_2_ incubator at 37°C according to the procedures described in the 2019 Brucellosis Diagnosis Criteria (WS269-2019). After 2 weeks, a single colony was inoculated. Then, it was incubated at 37°C for 24 hours, and the growth of the isolated strain was monitored. The *Brucella* PCR method was performed according to standard detection procedures. The reaction mixture consisted of 50 µL: 25 µL premix Taq, 0.2 mM F primer, 0.2 R primer, 3 µL DNA template from the sample, and nuclease-free water added to a volume of 50 µL. PCR premix solution was denatured at 94°C for 2 minutes, then 30 cycles including denaturation at 94°C (30 seconds), annealing at 55°C (40 seconds), and primer extension at 72°C (1 minute).

### Design and synthesis of guide RNA and MCDA primers

According to the principle of MCDA amplification, a total of 10 primers, including two displacement primers (F1 and F2), 6 amplification primers (C1, C2, R1, R2, D1, and D2), and 2 cross primers (CP1 and CP2) targeting *Bcsp31* and *BMEII0466* genes were designed using Primer Premier software (version 5.0). Using BLAST software (basic local alignment search tool) to analyze and screen the designed primers. Based on the principle of CRISPR/Cas12b reaction, a gRNA sequence was designed to guide Cas12b protein to undergo *trans* cleavage. All primers and gRNAs used in the experiment were synthesized by Tianyi Huiyuan Biotechnology Co., Ltd. (Beijing, China). The details of MCDA primers, gRNA position, and ssDNA reporter were shown in Fig. S1; Table S1.

### Preparation of DNA templates

The plasmid of *Bcsp31* and *BMEII0466* genes were synthesized by Tianyi Huiyuan Biotechnology Co., Ltd. (Beijing, China) (GenBank accession no. M20404.1 and GenBank: AE008918.1, respectively). The plasmid quantification was carried out using the Qubit4 Fluorometer system, and plasmid was diluted from a concentration of 2.0 × 10^5^ copies/μL to 2.0 × 10^−2^ copies/μL, which was used for optimizing reaction temperature and testing sensitivity of assays.

According to the instructions of the QIAamp DNA Mini kit, 200 µL of samples was extracted from each collection tube and 25 µL of proteinase K was added. Then pulse vortex for 15 seconds to ensure thorough mixing of the sample. Subsequently, added buffer solution and mixed the sample thoroughly. DNA was eluted from QIAamp MinElute, and the final volume of elution buffer was 100 µL.

### The CRISPR-MCDA-LFB pre-amplification reaction

The pre-amplification was performed in a 25 µL reaction volume. The reaction mixture included 0.1 µL displacement primers F1 and F2 (final concentration: 0.4 µM), 0.2 µL amplification primers C1, C2, R1, R2, D1, and D2 (final concentration: 0.8 µM), and 0.4 µL cross primers CP1 and CP2 (final concentration: 1.6 µM), 2.5 µL 10 × reaction buffer, 1.4 µL dNTP (dATP, dUTP, dGTP, dCTP, 25 nM), 1 μL MgSO_4_, 1 µL Bst 8.0 DNA polymerase (40U), 1 µL AUDG (100U), appropriate amount of DNA template (1.5 µL from pure culture and plasmids or 3 µL extracted from clinical samples), and DW (Double water) was supplemented to 25 µL (1 µL MG chromogenic reagents only used for confirming experiments). The MCDA reaction was first carried out at 37°C for 10 minutes, followed by 66°C for 50 minutes. Three different monitoring methods were used to confirm the reliability and specificity of the MCDA assay: LA-500, agarose gel electrophoresis, and MG chromogenic reagents.

### The CRISPR/Cas12b-mediated *trans* cleavage detection

The CRISPR/Cas12b-gRNA detection system (20 µL) consisted of 10 µL 2 × buffer, 4 µL Cas12b-gRNA complex, 1 µL ssDNA probe (5′-FAM-TTTTTTTT-Biotin-3', 100 nM; 5′-FAM-TTTTTTTT-BHQ1-3', 50 µM), 1 µL MCDA amplicon, and 4 µL nuclease-free water. The Cas12b gRNA complex was formed by pre-incubating 10 µM Cas12b nuclease and 10 µM gRNA in 1 × reaction buffer at 37°C for 10 minutes. Then, the reaction temperature was conducted at 48°C for 30 minutes, followed by RTF instrument or UV visual detection (with a positive reaction indicated by bright green fluorescence and a negative reaction by no color change), and LFB was used to verify the results.

### The nanoparticle-based lateral flow biosensor detection

The reaction was incubated at a constant temperature of 48°C for 20–30 minutes. Then, 30 µL RNase-free ddH_2_O was added to the reaction products before inserting the test strips and incubating for 5 minutes at room temperature. The results were read by visual inspection within 5 minutes of incubation. All of the reactions were repeated three times. When the probes labeled with FAM and Biotin in the CRISPR system were not cleaved, Biotin was fully captured at the CL, and when the probe was fully cleaved, FAM was captured at the TL. When the probe was not fully cleaved, it was captured at both the CL and the TL, and only the positive sample made the CRISPR system to activate the cleavage probe (Fig. S2).

### Optimization of conditions for CRISPR-MCDA-LFB detection

To achieve optimal detection efficiency, real-time turbidity was used to optimize the temperature (61°C−69°C, 1°C interval) and time (40–60 minutes, 10 minutes interval) of MCDA pre-amplification. The RTF instrument was then used to optimize the reaction cutting efficiency. Additionally, to determine the optimal reaction time, different times (1–30 with 5 minutes intervals) were tested. The results of CRISPR-MCDA assay were validated using RTF detector and LFB.

### The sensitivity and specificity of CRISPR-MCDA-LFB assay

For sensitivity evaluation of CRISPR-MCDA-LFB assay, serial dilutions of plasmid were prepared to cover a range from 2.0 × 10^5^ copies/µL to 2.0 × 10^−2^ copies/µL (2.0 × 10^5^, 2.0 × 10^4^, 2.0 × 10^3^, 2.0 × 10^2^, 2.0 × 10^1^, 2.0 × 10^0^, 2.0 × 10^−1^, 2.0 × 10^−2^). The reaction method follows the previously established reaction system, adding 1.5 µL of template DNA with different copy numbers to the system. The results of trans-cleavage were validated using the RTF instrument, UV visualization, and LFB methods.

The specificity of the CRISPR-MCDA-LFB method was evaluated using 28 strains of bacteria, including 7 *Brucella* strains (2 *B. abortus*, 3 *B. melitensis*, 2 *B. suis*) and 21 non-*Brucella* strains. According to the previously established method, adding 1.5 µL of DNA template to the reaction. The experimental results were validated by LFB. The information was shown in Table S2 and S3.

### The practicability of CRISPR-MCDA-LFB for clinical sample

A total of 64 body fluid samples including 54 whole blood, 9 serum, and 1 cerebrospinal fluid (it was collected on a cotton swab) were obtained from routine testing of *Brucella* by the Guizhou Provincial Center for Disease Control and Prevention to evaluate the practicability of the CRISPR-MCDA -LFB assay. The samples were collected from patients with positive antibodies in the initial screening. After centrifugation and mixing, 200 µL of each sample was transferred to an EP tube. The DNA was extracted strictly following the protocol of the QIAamp MinElute Virus Spin Kit (QIAGEN). Notably, for cerebrospinal fluid samples, 200 µL of 0.9% NaCl solution was added to EP tubes containing cotton swabs. The swabs were repeatedly squeezed, dissolved, and diluted approximately 20 times. After centrifugation, the bottom layer and precipitate were collected for nucleic acid extraction. The assay employed a bacterial culture as the gold standard and compared it with the *Brucella* PCR detection method to evaluate the practicality of CRISPR-MCDA-LFB.

## RESULTS

### The mechanism of the CRISPR-MCDA-LFB assay

**Fig 1:**
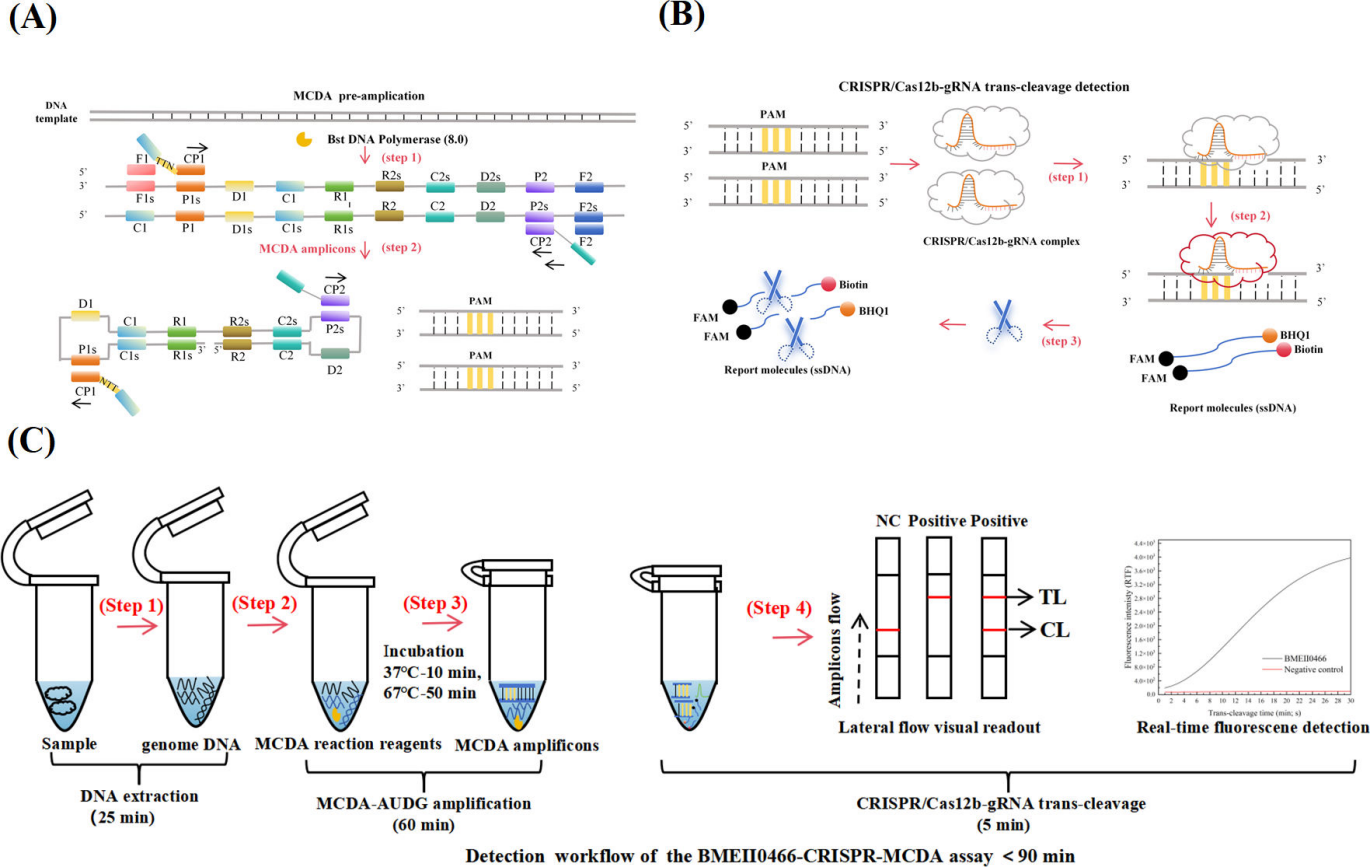
Schematic illustration of CRISPR/Cas12b-based multiplex cross displacement amplification detection assay (CRISPR-MCDA-LFB). (**A**) A description of the principle of MCDA pre-amplification. (**B**) The trans-cleavage mechanism of Cas12b-gRNA (guide RNA) complex. (**C**) Outline of the CRISPR-MCDA-LFB assay workflow, including extraction of the DNA template, MCDA pre-amplification, the trans-cleavage of CRISPR-Cas12b, and the final verification of the reaction results with LFB or RTF instrument.

The reaction mechanism of CRISPR-MCDA-LFB detection, as depicted in Fig. 1, was composed of MCDA pre-amplification and CRISPR/Cas12b mediated trans-cleavage detection. First, as shown in Fig. 1A, the MCDA pre-amplification reaction was performed. DNA extracted from the samples was used as the amplification template. Specific primers bound to the target region, and Bst 8.0 DNA polymerase was used to exponentially amplify the DNA, generating a large number of MCDA amplicons containing PAM sites. Next, the CRISPR/Cas12b gRNA system specifically binds to the MCDA amplicon, activating trans-cleavage activity. This enables the indiscriminate, non-specific cleavage of ssDNA probes labeled with Biotin and quenchers (BHQ1). This step represents the second stage of the CRISPR-MCDA-LFB assay: the trans-cleavage reaction (Fig. 1B). Finally, the results of the trans-cleavage reaction were validated using both the LFB and RTF instrument. The overall detection process of CRISPR-MCDA-LFB was shown in Fig. 1C, which included DNA template extraction (25 minutes), the MCDA-AUDG pre-amplification reaction (60 minutes), and CRISPR/Cas12b-based detection (48°C for 5 minutes). The entire process could be completed within 90 minutes.

### Confirmation test of CRISPR-MCDA-LFB assay

**Fig 2:**
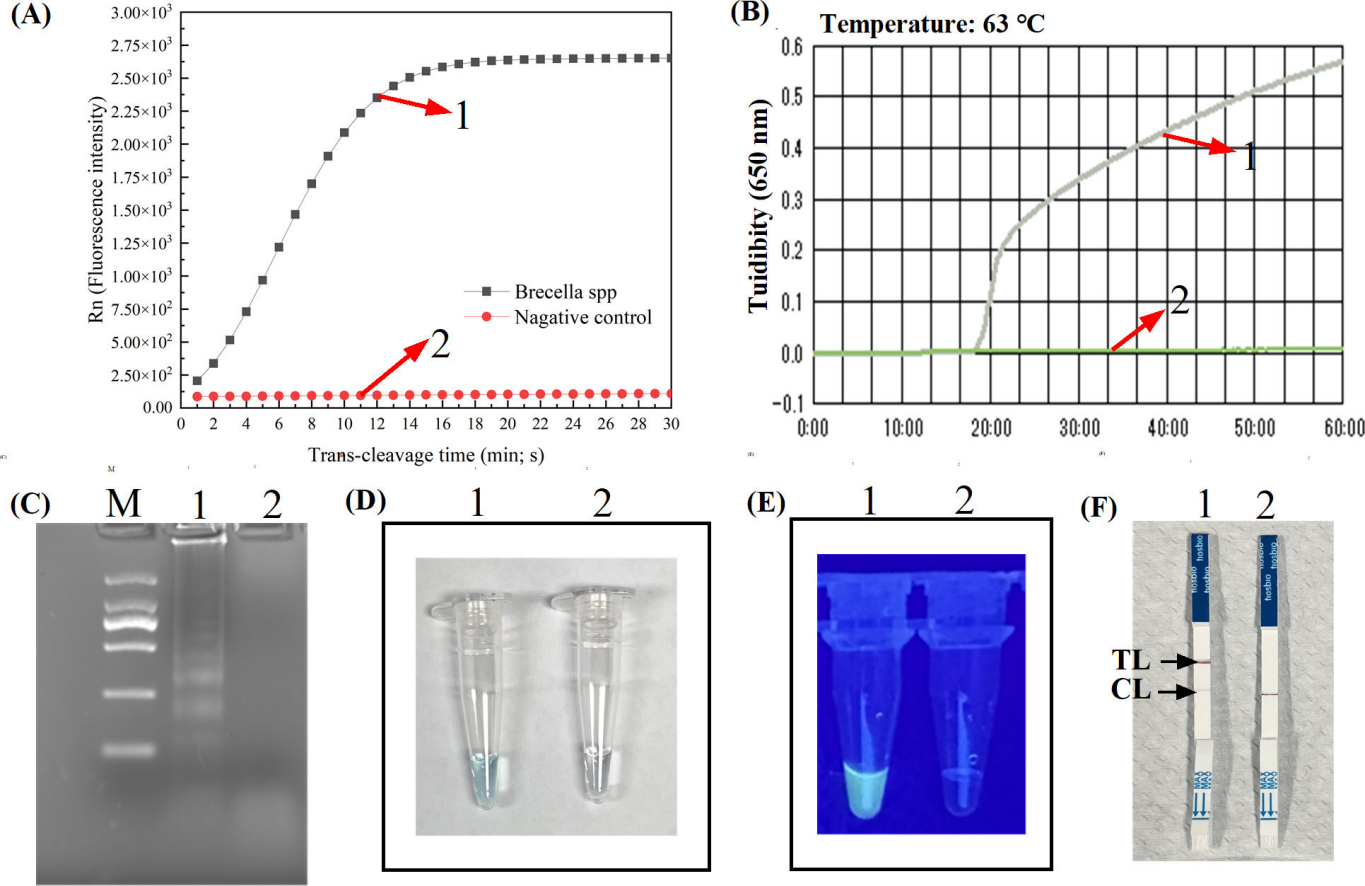
Confirmation tests for MCDA-*Bcsp31* pre-amplification and CRISPR-MCDA-LFB assays. There were two main steps, including the verification of the MCDA pre-amplification products (**B–D**) and the confirmation of the trans-cleavage detection (**A, E, and F**). MCDA amplicons were verified by real-time turbidity (**B**), 1.5% agarose gel electrophoresis (**C**), and MG visual indicator (**D**). The Cas12b/gRNA-mediated trans-cleavage was then confirmed by real-time fluorescence analysis (**A**), UV visualization detection (**E**), and LFB (**F**). No. 1 was positive and No. 2 was negative. CRISPR, clustered regularly interspaced short palindromic repeats; MCDA, multiple cross displacement amplification. MG, malachite green; CRISPR/Cas12b, clustered regularly interspaced short palindromic repeats (CRISPR)/CRISPR-associated 12b protein; MCDA, multiple cross displacement amplification; gRNA, guide RNA; LFB, nanoparticle-based lateral flow biosensor.

To confirm the feasibility of CRISPR-MCDA-LFB, LA-500, 1.5% agarose gel electrophoresis, and MG visual indicators were used to verify MCDA pre-amplification results. The turbidity analysis results showed that positive specimens exhibited a robust signal, whereas negative specimens lacked any discernible information (Fig. 2B). In gel electrophoresis analysis, the positive group amplified to produce a large number of products. Consistent with the MG reagent result, the positive result was blue and the negative result was colorless (Fig. 2C and D). All these results indicated the amplification effect of MCDA primers was robust. Then, Cas12b-gRNA mediated detection was then validated by RTF detector, UV visualization and LFB. The RTF analysis results showed that positive sample exhibited a robust signal, proving the high cutting efficiency of Cas12b (Fig. 2A). In LFB, the positive results showed red lines for both CL and TL, and the UV visualization results also demonstrated the usefulness of this primer (Fig. 2F and E). The CRISPR-MCDA-LFB of *B. melitensis* experimental results were shown in Fig. S4, which verified the effectiveness of the primers and gRNA used in the experiment.

### Optimal reaction conditions of the CRISPR-MCDA-LFB assay

**Fig 3:**
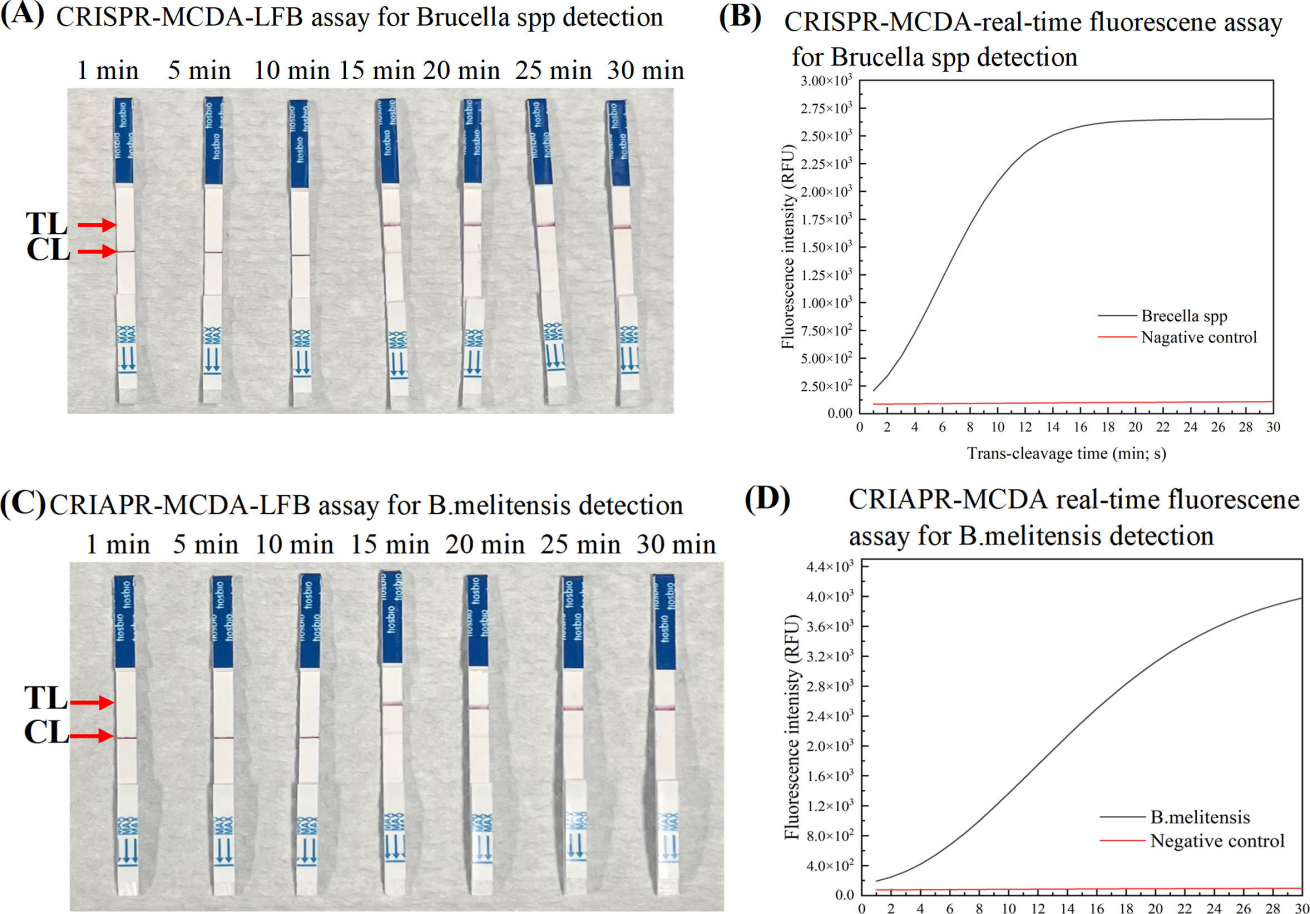
Optimal reaction time for CRISPR-MCDA-LFB (**A**) (*Bcsp31* detection) and (**C**) (*BMEII0466* detection). LFB was used for reporting the CRISPR/Cas12b-gRNA trans-cleavage results. A significant signal at the TL and a weak signal at the CL appeared on the biosensor within 15 minutes, indicating that the ssDNA reporter molecule was sufficiently cleaved. (**B**) (*Bcsp31* detection) and (**D**) (*BMEII0466* detection). The RTF instrument was used for reporting the CRISPR/Cas12b-gRNA cleavage results and further confirming the LFB analysis. CRISPR, clustered regularly interspaced short palindromic repeats; LFB, nanoparticle-based lateral flow biosensor; gRNA, guide RNA; RTF, real-time fluorescence.

In the optimization experiment, according to the previous reaction system, *Bcsp31* and *BMEII0466* plasmids DNA templates with a concentration of 2.0 × 10^7^ copies/μL were added. The results indicated that 67°C was the optimal reaction temperature and 50 minutes was the optimal amplification time for the MCDA pre-amplification step. Next, the reaction time for Cas12b-gRNA detection was analyzed with LFB and RTF instrument. The visual signals were observed within 5 minutes on LFB, and fluorescence signals were monitored within 1 minute by the RTF instrument (Fig. 3).

### The sensitivity and specificity of CRISPR-MCDA-LFB assay

**Fig 4:**
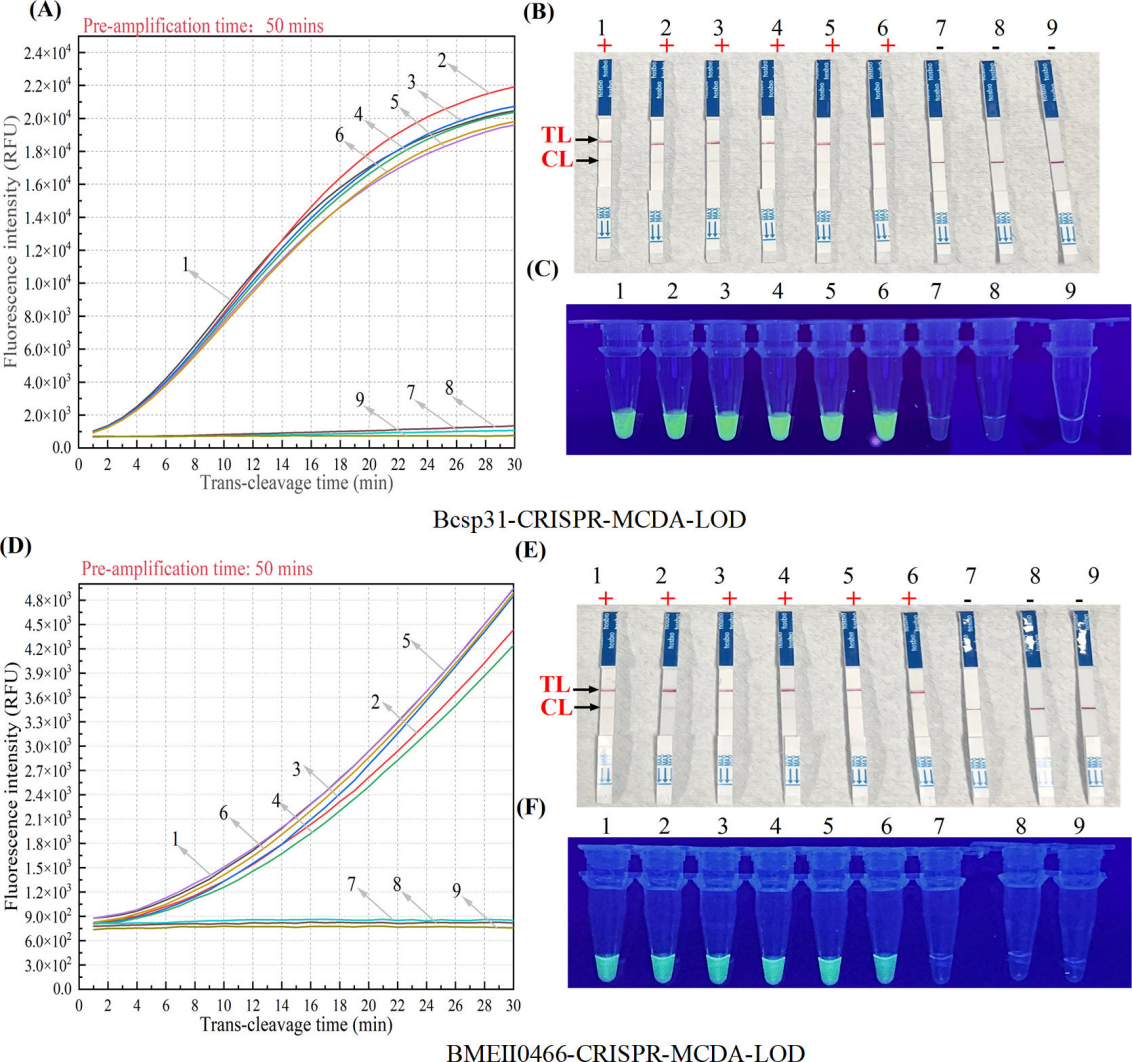
Sensitivity of the CRISPR-MCDA-LFB assay. (**A**)/(**D**) RTF instrument was reported on CRISPR-MCDA detection results. (**B**)/(**E**) The CRISPR-MCDA results were validated by LFB. (**C**)/(**F**) The CRISPR-MCDA results were visualized and detected under ultraviolet light. (**A**)–(**C**) were the validation results of the LOD for *Brucella* genus, while (**D**)–(**F**) were the validation results of the LOD for *B. melitensis*. Dilute genomic DNA as a positive template to validate the LOD of CRISPR-MCDA detection (2.0 × 10^5^ copies/μL, 2.0 × 10^4^ copies/μL, 2.0 × 10^3^ copies/μL, 2.0 × 10^2^ copies/μL, 2.0 × 10^1^ copies/μL, 2.0 × 10°copies/μL, 2.0 × 10^−1^ copies/μL, 2.0 × 10^−2^ copies/μL). The DNA templates corresponding to signals (**A**)(**D**)/ biosensor (**B**) (**E**)/ tubes (**C**)(**F**) 1–8 range from 2.0 × 10^5^ copies/μL to 2.0 × 10^−2^ copies/μL. The 9 correspond to blank control (nuclease-free water). The minimum concentration (2 copies/μL) of *Brucella* genus and *B. melitensis* genomic DNA that can be detected by real-time fluorescence, LFB, and visualization detection was consistent. LFB, nanoparticle-based lateral flow biosensor; LOD, limit of detection; RTF, real-time fluorescence.

**Fig 5:**
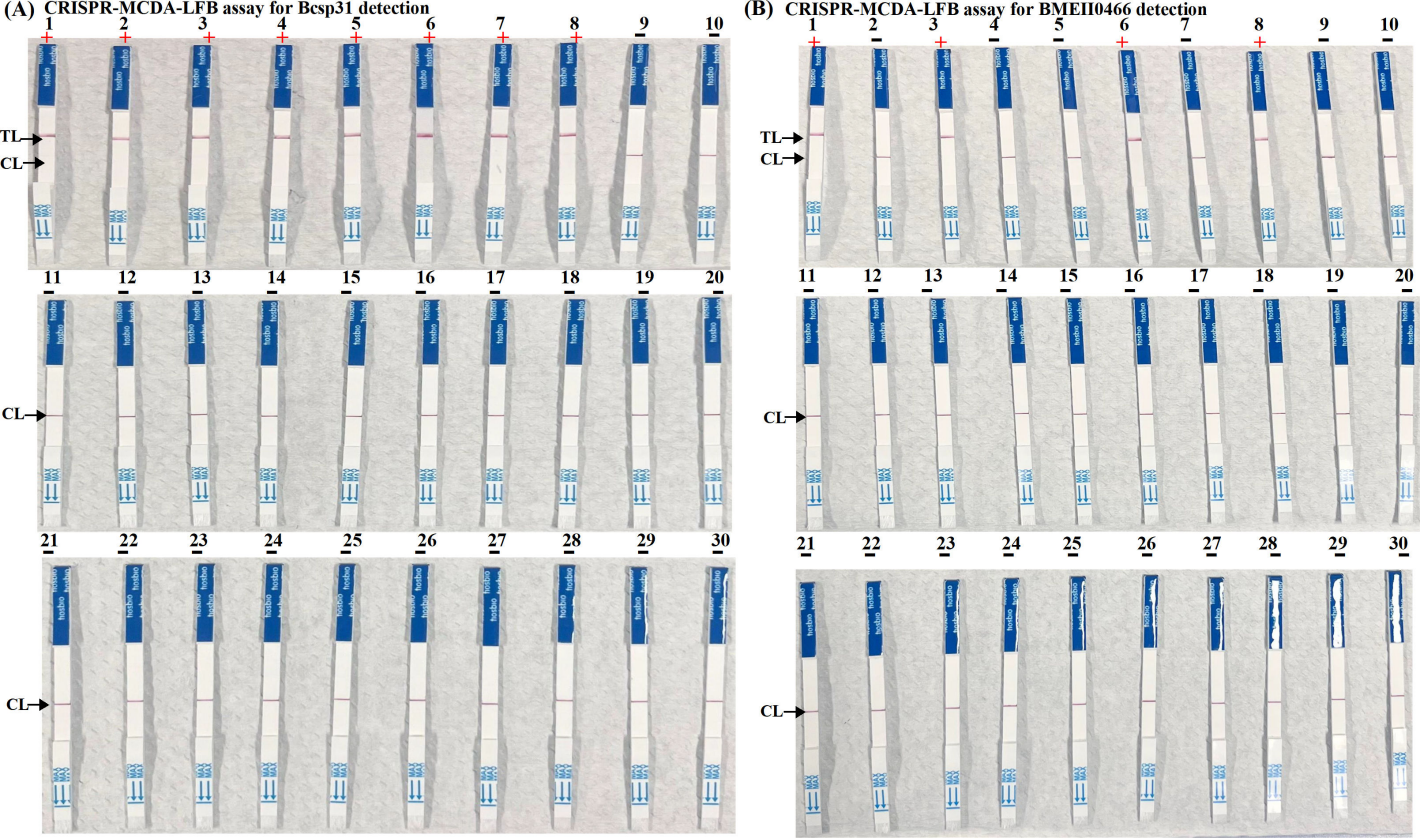
Specificity of the CRISPR-MCDA-LFB assay. (**A**) The specificity of the CRISPR-MCDA assay for *Brucella* genus detection. Biosensor 1, *Bcsp31* plasmid; Biosensors 2 to 8 *B. abortus* A19 (GZCDC); *B. melitensis* M5 (GZCDC), *B. suis* S2 (GZCDC), *B. abortus* 544 (NCTC 10093), *B. melitensis* 16M (NCTC 10094), *B. suis* 1330 (NCTC 10316), *B. melitensis* Ether (NCTC 10509), Biosensors 9 to 30 *Streptococcus pneumoniae* (GZCDC), *Pseudomonas aeruginosa* (GZCDC), *Mycobacterium tuberculosis* (GZCDC), *Staphylococcus aureus* (GZCDC), *Listeria monocytogenes* (GZCDC), *Haemophilus influenzae* (GZCDC), *Bacillus anthracis* (GZCDC), *Klebsiella pneumoniae* (GZCDC), *Orientia tsutsugamushi* (GZCDC), *Neisseria meningitidis* (GZCDC), Human Cytomegalovirus (GZCDC), *Mycobacterium leprae* (GZCDC), *Shigella sonnei* (GZCDC), *Salmonella* spp. (GZCDC), *Streptococcus suis* (GZCDC), blank control (nuclease-free water). CL, control line; TL, test line; “+”, positive; “−”, negative. (**B**) The specificity of the CRISPR-MCDA assay for *B. melitensis* detection. Biosensor 1, *BMEII0466* plasmid; Biosensors 2 to 8 *B. abortus* A19 (GZCDC); *B. melitensis* M5 (GZCDC), *B. suis* S2 (GZCDC), *B. abortus* 544 (NCTC 10093), *B. melitensis* 16M (NCTC 10094), *B. suis* 1330(NCTC 10316), *B. melitensis* Ether (NCTC 10509), Biosensors 9 to 30 *Streptococcus pneumoniae* (GZCDC), *Pseudomonas aeruginosa* (GZCDC), *Mycobacterium tuberculosis* (GZCDC), *Staphylococcus aureus* (GZCDC), *Listeria monocytogenes* (GZCDC), *Haemophilus influenzae* (GZCDC), *Bacillus anthracis* (GZCDC), *Klebsiella pneumoniae* (GZCDC), *Orientia tsutsugamushi* (GZCDC), *Neisseria meningitidis* (GZCDC), Human Cytomegalovirus (GZCDC), *Mycobacterium leprae* (GZCDC), *Shigella sonnei* (GZCDC), *Salmonella* spp. (GZCDC), *Streptococcus suis* (GZCDC), blank control (nuclease-free water). GZCDC, Guizhou Provincial Center for Disease Control and Prevention. CL, control line; TL, test line; “+”, positive; “−”, negative.

To verify the sensitivity of the CRISPR-MCDA-LFB method, we used the RTF instrument, UV visualization, and LFB, all of which exhibited a detection limit of 2 copies/μL, and the results of the three detection methods were consistent (Fig. 4). The CRISPR-MCDA-LFB specificity assay results showed that the primer have no cross-react with non-*Brucella* bacteria. The CRISPR-MCDA-LFB*-B. melitensis* results showed that only *Brucella* isolates from *B. melitensis* were positive, while other strains tested negative. The results of RTF detection and LFB detection were consistent in the specificity assay. These results demonstrated the high specificity of CRISPR-MCDA-LFB assay (Fig. 5; Tables S2 and S3).

### The practicability of CRISPR-MCDA-LFB for clinical sample

According to the results of bacterial culture, five samples were confirmed to be infected with *Brucella* including one cerebrospinal fluid and four whole blood samples. The 9 serum and 50 whole blood samples were determined to be non-*Brucella* infections. As shown in Table 1, the CRISPR-MCDA-LFB-*Bcsp31* detection results were consistent with bacterial culture and PCR-*Bcsp31* detection (5/5) in clinical sample. However, only 3 of 5 *Brucella* genus positive samples were *B. melitensis* species positive detected by both PCR-*B. melitensis* and CRISPR-MCDA-LFB-*B. melitensis*.

**TABLE 1:** Detection results of CRISPR-MCDA-LFB for clinical samples^*a*^

Method	Bacterial culture	
	Positive (*n* = 5)	Negative (*n* = 59)
CRISPR -MCDA-LFB-*Bcsp31*		
Positive	5	0
Negative	0	59
PCR-*Bcsp31*		
Positive	5	0
Negative	0	59
CRISPR-MCDA-LFB-*BMEII0466*		
Positive	3	0
Negative	2	59
PCR-*BMEII0466*		
Positive	3	0
Negative	2	59

^*a*^ CRISPR, clustered regularly interspaced short palindromic repeats; MCDA, multiple cross displacement amplification; LFB, nanoparticle-based lateral flow biosensor. Bcsp31, the specific gene of *Brucella genus*; *BMEII0466*, the specific gene of *B. melitensis*.

## DISCUSSION

Brucellosis caused by *Brucella* spp., which can seriously endanger human health, hinder the development of animal husbandry and related industries, and affect economic growth. WHO has listed brucellosis as one of the easily overlooked zoonotic diseases that threatens human and animal health (27, 28). In the majority of laboratory acquired brucellosis cases, the serotype was reported as *B. melitensis* (8). Therefore, the development of rapid, reliable, sensitive, and easily accessible identification methods for *Brucella* genus and *B. melitensis* are crucial for the diagnosis of brucellosis. Although bacterial culture and standard tube agglutination tests are still canonical methods for detecting brucellosis, the inherent limits like time-consuming, low sensitivity, and risk of infection for laboratory operators make it difficult to meet the above requirements (10). It cannot be denied that these rapid detection techniques, including PCR and PCR-based detection, are more sensitive than traditional culture and more specific than serological tests (6, 9). However, specialized testing instruments have hindered its further clinical application.

In recent years, the nucleic acid detection technology based on the CRISPR/Cas system is considered a desirable molecular detection scheme (29). In the CRISPR/Cas system, Cas12b has high cleavage activity at higher temperatures (37–60°C), which can be used for rapid cleavage of target nucleic acids (21, 24, 25). Here, we designed CRISPR/Cas12b combined with MCDA for the first time to detect *Brucella* (*Bcsp31*) and *B. melitensis* (*BMEII0466*) simultaneously. The specific genes, as common molecular target for *Brucella* detection in previous studies, were used to design the specific MCDA primers and gRNA in the CRISPR-MCDA (30). Meanwhile, the specificity of the CRISPR-MCDA-LFB method has been strongly confirmed in the *Brucella* strains and other non-*Brucella* strains. Using LFB, the results can be validated within 5 minutes, shortening the time for amplicon validation. In addition, the reduced requirements for amplification reaction equipment make it suitable for a broader range of applications.

However, the MCDA technique is highly susceptible to carryover contamination due to its extreme sensitivity (16, 19, 31). To address this issue, we incorporated AUDG and replaced dTTP with dUTP in all reaction products. The reaction mixture was incubated with AUDG at 37°C for 10 minutes to eliminate residual contamination. As shown in Fig. S3, the results showed the CRISPR-MCDA-LFB-*Bcsp31* can eliminate pollution concentrations of 1.0 × 10^−13^ g/µL, while CRISPR-MCDA-LFB-*BMEII0466* could eliminate pollution concentrations of 1.0 × 10^−12^ g/µL. The AUDG showed excellent effectiveness in eliminating residual contamination experiments. In the CRISPR-MCDA-LFB system, different detection methods were used for MCDA product and *trans*-cleavage validation, and results demonstrated the feasibility of the primers used in the study (Fig. 2; Fig. S4). In addition, in the optimization experiment of trans-cleavage time, the results indicated that amplification signals could be detected within 15 minutes on LFB (Fig. 3). Both RTF analysis and the LFB method can be used for CRISPR detection. The RTF analysis requires special instruments, while the LFB method is easy to operate and time-saving. In our method, using LFB to verify experimental results can more quickly identify *Brucella* in POC detection.

In this study, the CRISPR-MCDA-LFB detection showed high sensitivity and can detect genomic DNA at a minimum concentration of 2 copies/μL. In addition, RTF detection, LFB, and UV visualization detection consistently verified Cas12b-gRNA mediated *trans*-cleavage results (Fig. 4). The outstanding advantage of ultra sensitivity plays a crucial role in improving detection rates, especially in *B. melitensis* with low bacterial load. In addition, the CRISPR-MCDA-LFB-*Brucella* assay successfully identified all examined *Brucella* strains and 21 other non-*Brucella* strains. The CRISPR-MCDA-LFB-*B. melitensis* assay identified all examined *B. melitensis* strains and did not cross-reaction with other *Brucella* spp. (*B. abortus* and *B. suis*) and non-*Brucella* pathogens (Fig. 5). The experimental results indicated that the gene sequence designed in the experiment has strong specificity. To further confirm the sensitivity of CRISPR-MCDA-LFB detection in human fluid samples, we successfully applied CRISPR-MCDA-LFB to detect various clinical samples (Table 1). The clinical sample detection demonstrated that the *Brucella* genus positive rates of CRISPR-MCDA-LFB-*Brucella* were consistent with bacterial culture and PCR-*Brucella* assay. The results of the CRISPR-MCDA-LFB-*B. melitensis* method were consistent with the PCR-*B. melitensis* test, but 2 of 5 *Brucella* genus positive samples were *B. melitensis* species negative detected by both PCR-*B. melitensis* and CRISPR-MCDA-LFB-*B. melitensis*, which suggested that the 2 samples belong to other *Brucella* species. We are also considering other methods, such as whole-genome sequencing, to further identify the 2 *Brucella* strains isolated from the samples.

Although the current detection method had yield promising results, its one-pot method still needs further consideration. However, the integration of sample pretreatment and nucleic acid detection has some challenges: (i) although the one-pot method can achieve rapid detection, it may not meet the high-precision requirements of laboratory testing, because some low-concentration *Brucella* samples, the one-pot POC detection may not be effectively identified, resulting in missed diagnosis (32). (ii) In our study, the Cas system was incompatible with the isothermal amplification system, and the Cas12b will compete with the isothermal amplification enzyme and cleaves the target gene, leading to a decrease in the detection ability of this method (23, 33). Despite these challenges, we are actively working to optimize the reaction system and detection method, with the goal of establishing a CRISPR-MCDA-LFB one-step detection method for *Brucella*.

In conclusion, this report successfully designed and established CRISPR-MCDA-LFB technology for simultaneous detection of *Brucella* genus and *B. melitensis*. The method had high sensitivity and specificity, detecting as low as 2 copies/μL within 90 minutes. The CRISPR-MCDA-LFB-*Brucella* had no cross-reaction with other non-*Brucella* strains, and CRISPR-MCDA-LFB-*B. melitensis* did not cross-reaction with other *Brucella* spp. (*B. abortus* and *B. suis*) and non-*Brucella* pathogens. Moreover, the LFB detection results were used, and this method reduced detection time while eliminating the need for specialized instruments. Taken together, the CRISPR-MCDA-LFB detection method established in this study was a rapid, ultra-sensitive, highly specific, and readily available detection method, which can serve as a valuable/potential diagnostic tool for clinical brucellosis infection.

## Data Availability

All data sets generated for this research are available from the corresponding authors.

## SUPPLEMENTAL MATERIAL

Supplemental Figures jcm.01532-24-s0001.docx Figures S1 to S4.

Supplemental Tables jcm.01532-24-s0002.docx Tables S1 to S3.

ASM does not own the copyrights to Supplemental Material that may be linked to, or accessed through, an article. The authors have granted ASM a non-exclusive, world-wide license to publish the Supplemental Material files. Please contact the corresponding author directly for reuse.
