# Supplementary material for: Ultrasensitive and highly specific detection of the Brucella genus and B. melitensis by CRISPR/Cas12b‐multiple cross displacement amplification technique
Source: J Clin Microbiol. 2025 Apr 11;63(5):e01532-24. doi: 10.1128/jcm.01532-24 (PMC12077202; doi:10.1128/jcm.01532-24)
Supplement: Supplemental Figures — Figures S1 to S4. [file jcm.01532-24-s0001.docx]

**Supplementary Figures**


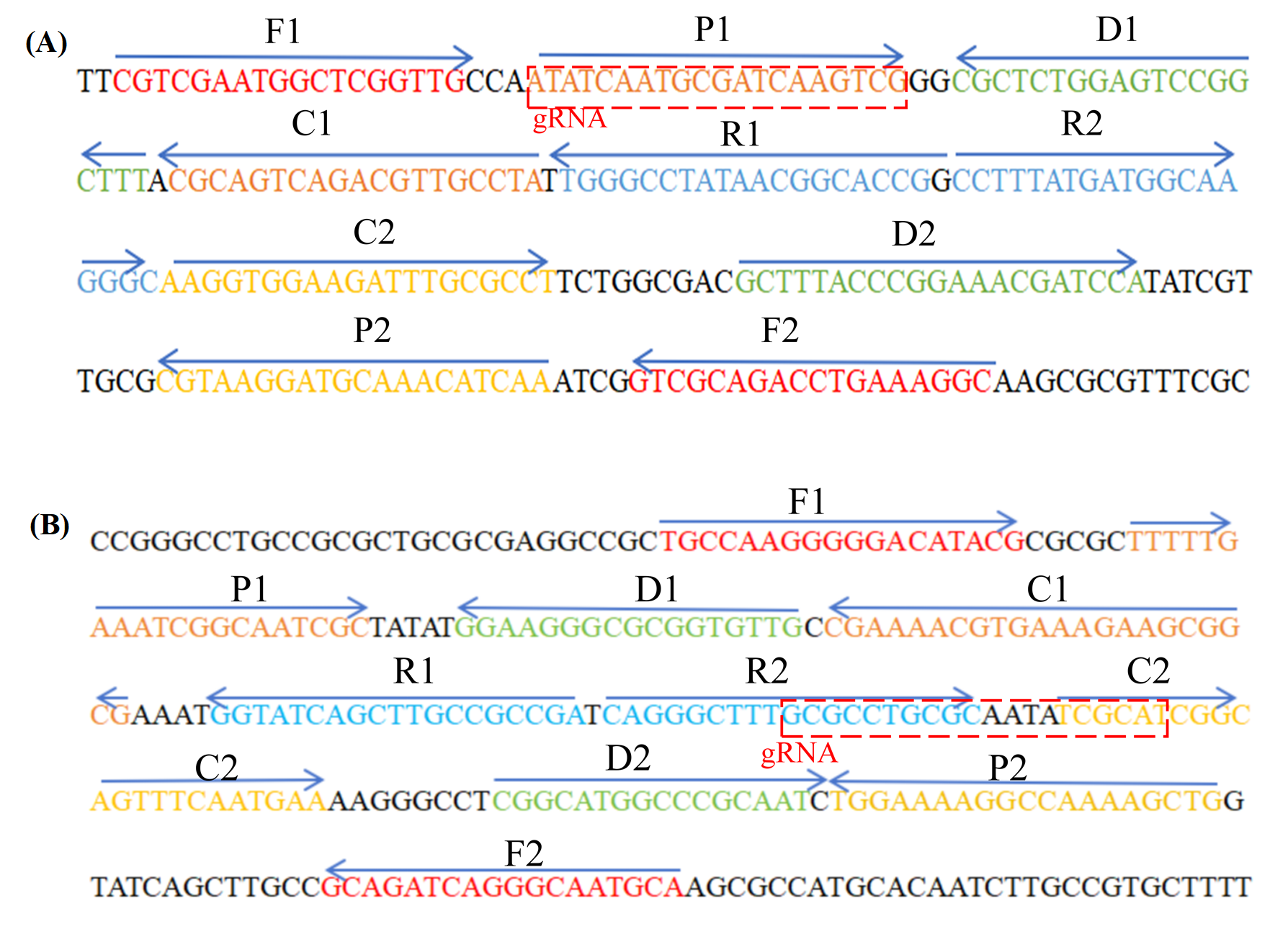


**Supplementary Figure S1.** **The location and sequence of MCDA primers and gRNA used in CRISPR- MCDA assay.** (A) was the DNA sequence of *Brucella*, and (B) was DNA sequence of *B. melitensis*. Primer sequences were shown in colored font, and the gRNA was displayed in red dashed boxes. The direction of arrows indicated the primer from 5′ to 3′. CRISPR: clustered regularly interspaced short palindromic repeats; MCDA: multiple cross displacement amplification; gRNA: guide RNA.


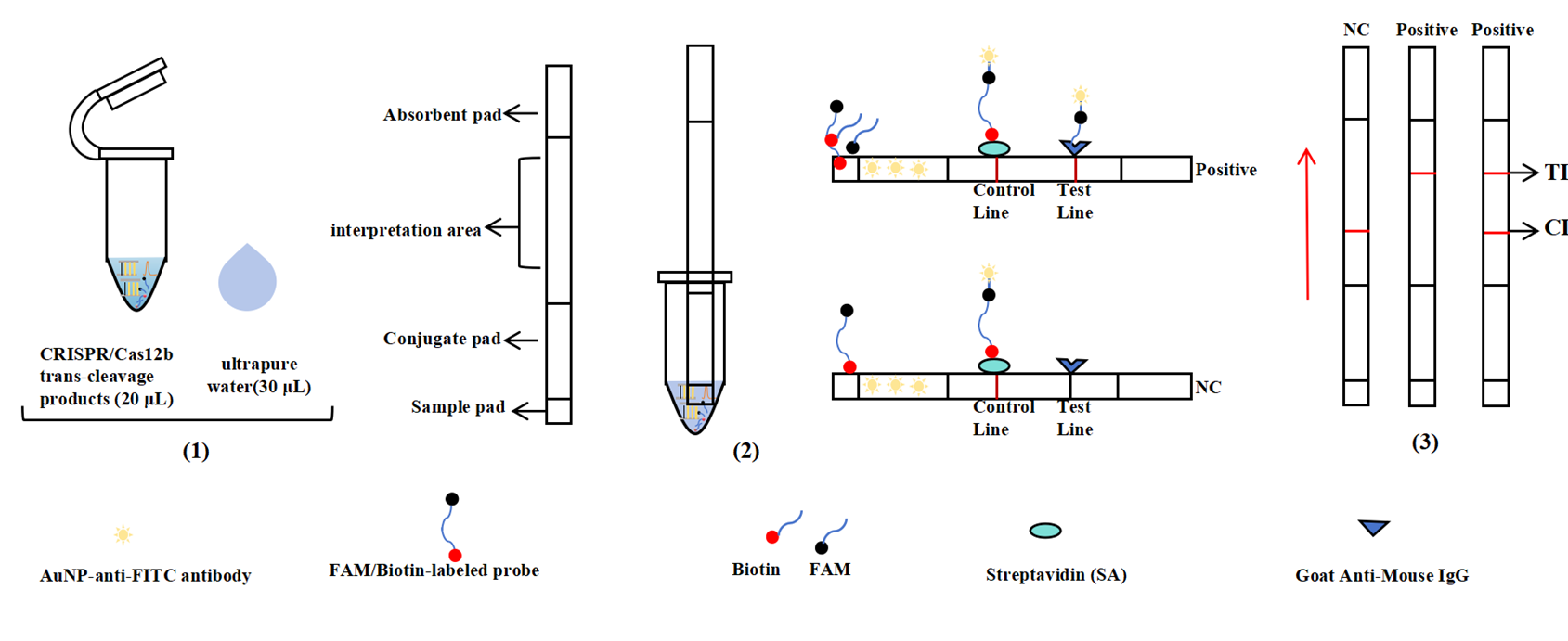


**Supplementary Figure S2.** **LFB visualization verification experiment**. Added 30 μL ultrapure water into 20 μL reaction mixing tube and mix well. (1). The LFB was inserted into the reaction tube and waited for 5 minutes. In positive samples, the ssDNA reporter molecule (5'- FAM-TTTTTTTT-Biotin-3') was trans-cleaved by activated CRISPR/Cas12b nuclease, so the FAM and Biotin were isolated. The FAM of the ssDNA reporter molecule captured to Goat Anti-Mouse IgG for visualization on TL. However, the negative results showed that the ssDNA reporter molecule was not cleaved and was specifically captured by SA on CL. (2, 3). CL: Control line; TL: Test line.


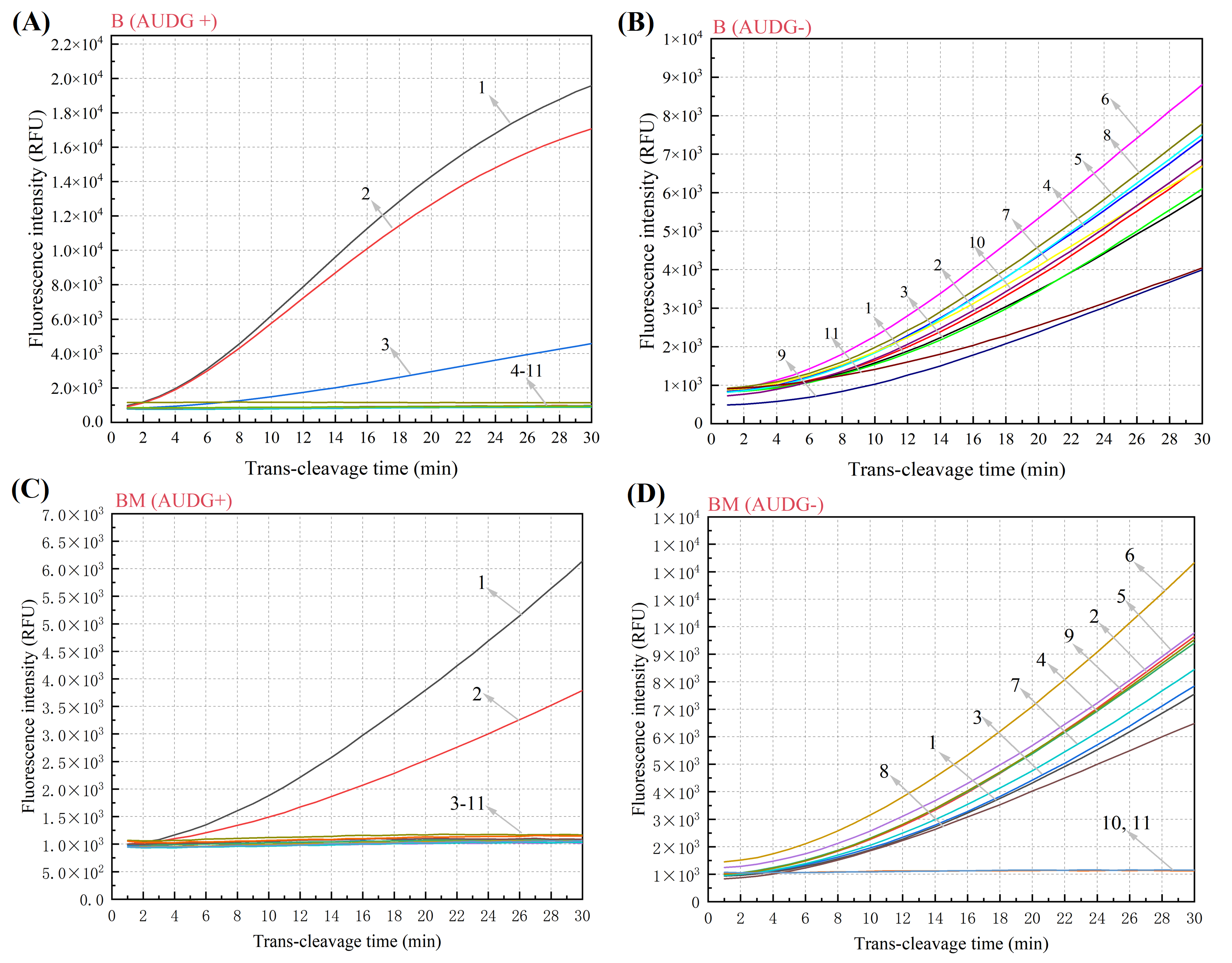


**Supplementary Figure S3.** **The assay of AUDG to remove false positive amplification.** The effect of AUDG was confirmed by detecting the detection limit (LOD) of simulated environmental pollution, and the detection sample concentration was from 1.0×10^-11^ g/μL to 1.0×10^-21^ g/μL. Then, the detection was based on CRISPR/Cas12b-gRNA combined with multi-crossover displacement amplification (MCDA) technology with the best-optimized conditions and system. Fig. (A) showed the detection result of CRISPR-MCDA-*Bcsp31* with the addition of AUDG, while Fig. (B) showed the LOD detection result without the addition of AUDG in the reaction tube. Fig. (C) showed the LOD of CRISPR-MCDA-*BMEII0466* with the addition of AUDG, and Fig. (D) showed the LOD without the addition of AUDG in the reaction. AUDG: antarctic thermal sensitive uracil-DNA-glycosylase; gRNA: guide RNA; *Bcsp31*; specific genes of *Brucella*; *BMEII0466*: The specific gene of *B.* *melitensis*.


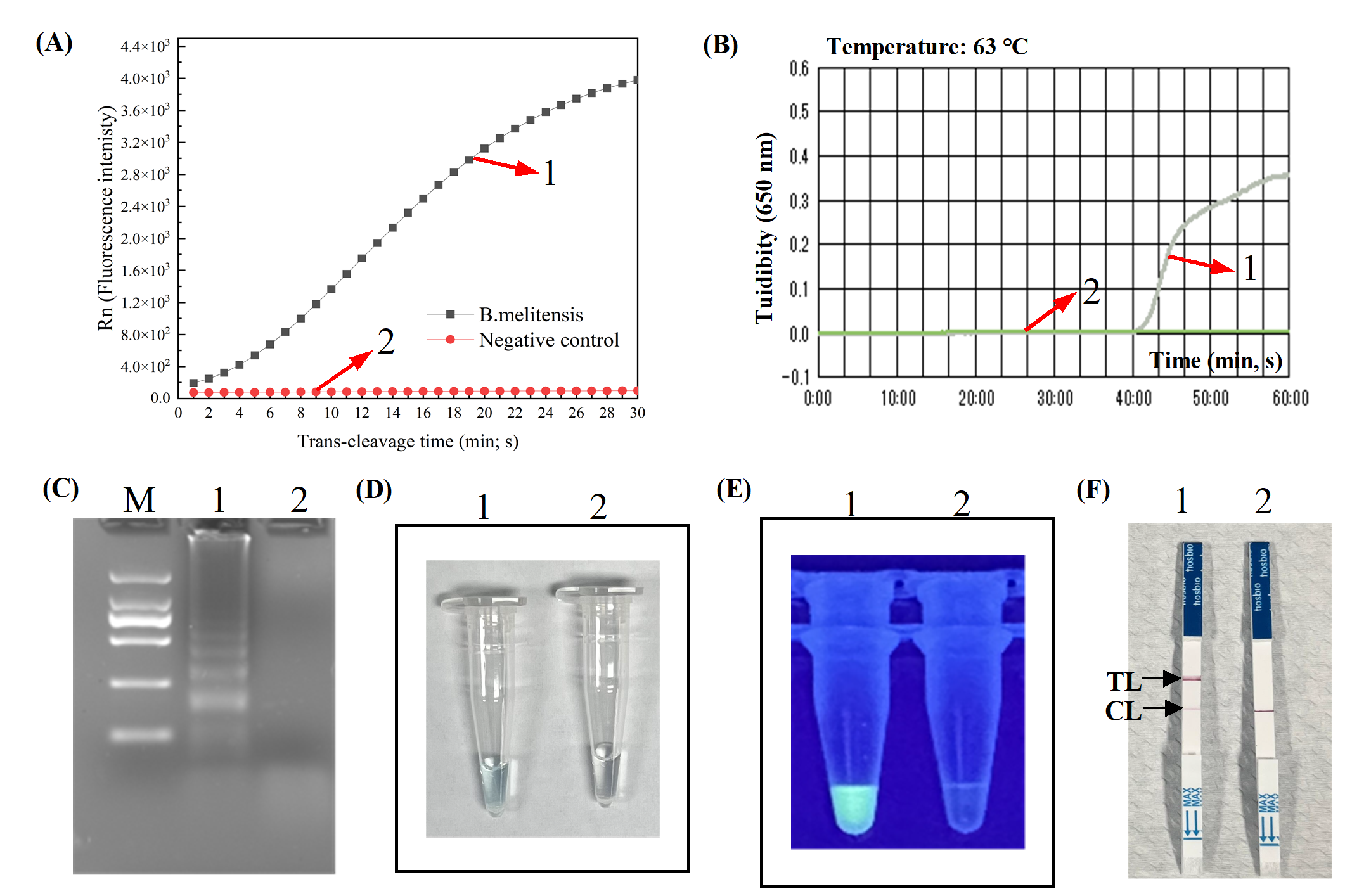


**Supplementary Figure S4. Confirmative tests for *BMEII0466* pre-amplification and Cas12b/gRNA-mediated trans-cleavage detection in CRISPR-MCDA assays.** There were two main steps, including the verification of the MCDA pre-amplification products (**B, C** and **D**) and the confirmation of the trans-cleavage detection (**A,** **E** and **F**). MCDA amplicons were verified by real-time turbidity (**B**), 1.5% agarose gel electrophoresis (**C**) and MG visual indicator (**D**). The Cas12b/gRNA-mediated trans- cleavage was then confirmed by real-time fluorescence analysis (**A**), visualization detection (**D**) and LFB (**F**). No.1 was positive and No.2 was negative. CRISPR: clustered regularly interspaced short palindromic repeats; MCDA: multiple cross displacement amplification. MG: malachite green; CRISPR/Cas12b: clustered regularly interspaced short palindromic repeats (CRISPR)/CRISPR-associated 12b protein; MCDA: multiple cross displacement amplification; gRNA: guide RNA; LFB: nanoparticle-based lateral flow biosensor.
