# Supplementary material for: Ultrasensitive and highly specific detection of the Brucella genus and B. melitensis by CRISPR/Cas12b‐multiple cross displacement amplification technique
Source: J Clin Microbiol. 2025 Apr 11;63(5):e01532-24. doi: 10.1128/jcm.01532-24 (PMC12077202; doi:10.1128/jcm.01532-24)
Supplement: Supplemental Tables — Tables S1 to S3. [file jcm.01532-24-s0002.docx]

**Supplementary Material**

**Supplementary Table S1.** The primers used in the current study

| Primer/gRNA^a^ | Sequence^b^ | Length^c^ |
| --- | --- | --- |
| *Bcsp31* |  |  |
| F1 | 5'-CGTCGAATGGCTCGGTTG-3' | 18 nt |
| F2 | 5'-GCCTTTCAGGTCTGCGAC-3' | 18 nt |
| CP1 | 5'-TAGGCAACGTCTGACTGCGTTTATATCAATGCGATCAAGTCG-3' | 42 nt |
| CP2 | 5'-AAGGTGGAAGATTTGCGCCTTTGATGTTTGCATCCTTACG-3' | 40 nt |
| C1 | 5'-TAGGCAACGTCTGACTGCGT-3' | 20 nt |
| C2 | 5'-AAGGTGGAAGATTTGCGCCT-3' | 20 nt |
| D1 | 5'-AAAGCCGGACTCCAGAGCG-3' | 19 nt |
| D2 | 5'-GCTTTACCCGGAAACGATCCA-3' | 21 nt |
| R1 | 5'-CGGTGCCGTTATAGGCCCA-3' | 19 nt |
| R2 | 5'-CCTTTATGATGGCAAGGGC-3' | 19 nt |
| gRNA | 5'-GUCUAGAGGACAGAAUUUUUCAACGGGUGUGCCAAUGGCCACUUUCCAGGUGGCAAAGCCCGUUGAGCUUCUCAAAUCUGAGAAGUGGCACAUAUCAAUGCGAUCAAGUCG-3' | 111 mer |
| F primer | 5'-CGTCGAATGGCTCGGTTG-3' | 18 nt |
| R primer | 5'-GCCTTTCAGGTCTGCGAC-3' | 18 nt |
| *BMEⅡ0466* |  |  |
| F1 | 5'-TGCCAAGGGGGACATACG-3' | 18 nt |
| F2 | 5'-TGCATTGCCCTGATCTGC-3' | 18 nt |
| CP1 | 5'-CGCCGCTTCTTTCACGTTTTCGTTTTTGAAATCGGCAATCGC-3' | 42 nt |
| CP2 | 5'-TCGCATCGGCAGTTTCAATGAACAGCTTTTGGCCTTTTCCA-3' | 41 nt |
| C1 | 5'-CGCCGCTTCTTTCACGTTTTCG-3' | 22 nt |
| C2 | 5'-TCGCATCGGCAGTTTCAATGAA-3' | 22 nt |
| D1 | 5'-CAACACCGCGCCCTTCC-3' | 17 nt |
| D2 | 5'-CGGCATGGCCCGCAAT-3' | 16 nt |
| R1 | 5'-TCGGCGGCAAGCTGATACC-3' | 19 nt |
| R2 | 5'-CAGGGCTTTGCGCCTGCGC-3' | 19 nt |
| gRNA | 5'-GUCUAGAGGACAGAAUUUUUCAACGGGUGUGCCAAUGGCCACUUUCCAGGUGGCAAAGCCCGUUGAGCUUCUCAAAUCUGAGAAGUGGCACGCGCCUGCGCAAUAUCGCAU-3' | 111 mer |
| Probe-1 | 5'-FAM-TTTTTTTT-BHQ1-3' | 8 nt |
| Probe-2 | 5'-FAM-TTTTTTTT-Biotin-3' | 8 nt |
| F primer | 5'-TGCCAAGGGGGACATACG-3' | 18 nt |
| R primer | 5'-TGCATTGCCCTGATCTGC-3' | 18 nt |

^a^ gRNA: guide RNA;

^b^ The primer was modified with a PAM site (TTC) in the linker region.

^c^ mer: monomeric unit; nt: nucleotide.

**Table S2.** The information of bacterial strains for *Bcsp31* detection.

| Bacteria | Strain no. (source  of strains) ^a^ | No. of  strains | CRISPR-MCDA-RTF (B) result ^b^ | CRISPR-MCDA-LFB (B)result ^b^ |
| --- | --- | --- | --- | --- |
| *B. abortus* | A19 (GZCDC) | 1 | P | P |
| *B. melitensis* | M5 (GZCDC) | 1 | P | P |
| *B. suis* | S2 (GZCDC) | 1 | P | P |
| *B. abortus* | 544 (NCTC 10093) | 1 | P | P |
| *B. melitensis* | 16M (NCTC10094) | 1 | P | P |
| *B. suis* | 1330(NCTC 10316) | 1 | P | P |
| *B. melitensis* | Ether (NCTC 10509) | 1 | P | P |
| *Streptococcus pneumoniae* | Isolated strains (GZCDC) | 1 | N | N |
| *Pseudomonas aeruginosa* | Isolated strains (GZCDC) | 1 | N | N |
| *Mycobacterium tuberculosis* | Isolated strains (GZCDC) | 1 | N | N |
| *Staphylococcus aureus* | Isolated strains (GZCDC) | 1 | N | N |
| *Listeria monocytogenes* | Isolated strains (GZCDC) | 1 | N | N |
| *Haemophilus influenzae* | Isolated strains (GZCDC) | 1 | N | N |
| *Bacillus anthracis* | Isolated strains (GZCDC) | 1 | N | N |
| *Klebsiella pneumoniae* | Isolated strains (GZCDC) | 1 | N | N |
| *Orientia tsutsugamushi* | Isolated strains (GZCDC) | 1 | N | N |
| *Neisseria meningitidis* | Isolated strains (GZCDC) | 1 | N | N |
| Human Cytomegalovirus | Isolated strains (GZCDC) | 1 | N | N |
| *Mycobacterium leprae* | Isolated strains (GZCDC) | 1 | N | N |
| *Shigella sonnei* | Isolated strains (GZCDC) | 1 | N | N |
| *Salmonella spp* | Isolated strains (GZCDC) | 4 | N | N |
| *Streptococcus suis* | Isolated strains (GZCDC) | 4 | N | N |
| Total |  | 28 |  |  |

^a^ GZCDC, Guizhou Provincial Center for Disease Control and Prevention; NCTC, National Collection of Type Cultures;

^b^ P, Positive; N, Negative; B: The specific gene *Bcsp31* of *Brucella genus*.; CRISPR: clustered regularly interspaced short palindromic repeats; MCDA: multiple cross displacement amplification; LFB: nanoparticle-based lateral flow biosensor; RTF: real-time fluorescence.

**Table S3****.** The information of bacterial strains for *BMEⅡ0466* detection.

| Bacteria | Strain no. (source  of strains) ^a^ | No. of  strains | CRISPR-MCDA-RTF(BM)result ^b^ | CRISPR-MCDA-LFB(BM)result ^b^ |
| --- | --- | --- | --- | --- |
| *B. abortus* | A19 (GZCDC) | 1 | N | N |
| *B. melitensis* | M5 (GZCDC) | 1 | P | P |
| *B. suis* | S2 (GZCDC) | 1 | N | N |
| *B. abortus* | 544 (NCTC 10093) | 1 | N | N |
| *B. melitensis* | 16M (NCTC10094) | 1 | P | P |
| *B. suis* | 1330(NCTC 10316) | 1 | N | N |
| *B. melitensis* | Ether (NCTC 10509) | 1 | P | P |
| *Streptococcus pneumoniae* | Isolated strains (GZCDC) | 1 | N | N |
| *Pseudomonas aeruginosa* | Isolated strains (GZCDC) | 1 | N | N |
| *Mycobacterium tuberculosis* | Isolated strains (GZCDC) | 1 | N | N |
| *Staphylococcus aureus* | Isolated strains (GZCDC) | 1 | N | N |
| *Listeria monocytogenes* | Isolated strains (GZCDC) | 1 | N | N |
| *Haemophilus influenzae* | Isolated strains (GZCDC) | 1 | N | N |
| *Bacillus anthracis* | Isolated strains (GZCDC) | 1 | N | N |
| *Klebsiella pneumoniae* | Isolated strains (GZCDC) | 1 | N | N |
| *Orientia tsutsugamushi* | Isolated strains (GZCDC) | 1 | N | N |
| *Neisseria meningitidis* | Isolated strains (GZCDC) | 1 | N | N |
| Human Cytomegalovirus | Isolated strains (GZCDC) | 1 | N | N |
| *Mycobacterium leprae* | Isolated strains (GZCDC) | 1 | N | N |
| *Shigella sonnei* | Isolated strains (GZCDC) | 1 | N | N |
| *Salmonella spp.* | Isolated strains (GZCDC) | 4 | N | N |
| *Streptococcus suis* | Isolated strains (GZCDC) | 4 | N | N |
| Total |  | 28 |  |  |

^a^ GZCDC, Guizhou Provincial Center for Disease Control and Prevention; NCTC, National Collection of Type Cultures;

^b^ P, Positive; N, Negative; BM: The specific gene *BMEII0466* of *B. melitensis*; CRISPR: clustered regularly interspaced short palindromic repeats; MCDA: multiple cross displacement amplification; LFB: nanoparticle-based lateral flow biosensor; RTF: real-time fluorescence.
